# Multi-trait assessment of *Meloidogyne incognita* resistance in developed *Cucumis* spp. inbred lines: horticultural, physiological, biochemical, and histological insights

**DOI:** 10.1186/s12870-026-09586-4

**Published:** 2026-08-05

**Authors:** Eman El-Remaly, Ahmed ASA El-Eslamboly

**Affiliations:** 1 Cross-Pollinated Vegetables Research Department, Horticultural Research Institute, Agricultural Research Center, Giza, Egypt; 2 Vegetable Crops Production Under Protected Cultivation Research Department, Horticultural Research Institute, Agricultural Research Center, Giza, Egypt

**Keywords:** *Cucumis metuliferus*, *Cucumis sativus* var *hardwickii*, *Meloidogyne incognita*, Coefficient of variation, Antioxidant enzymes, Phytohormones

## Abstract

This study aimed to develop nematode-resistant inbred lines from *Cucumis sativus*, *C. sativus* var. *hardwickii*, and *C. metuliferus*, and to elucidate their resistance mechanisms against *Meloidogyne incognita*. Three parental genotypes were self-pollinated across five generations, with simultaneous selection for superior horticultural traits and nematode resistance. Resistance levels were assessed based on root gall index, gall number, egg mass, and egg count per root system. To investigate the underlying mechanisms, histological, biochemical, and molecular analyses were conducted, focusing on salicylic acid (SA) content and the expression of SA pathway genes (*PR1-1A*, *PR3*, and *LOX1*). Based on the coefficient of variation (CV%) for horticultural traits, three inbred lines were selected from *C. sativus* (Cs), three from *C. sativus* var. *hardwickii* (Ch), and five from *C. metuliferus* (Cm). Among these, Cs-L1, Ch-L2, Cm-L1, and Cm-L2 exhibited superior horticultural traits, whereas Cs-L1, Ch-L3, Cm-L4, and Cm-L5 showed the lowest nematode infection parameters. Histological examination revealed localized necrosis and cellular degeneration in the resistant lines, suggesting a hypersensitive response (HR)-like mechanism that likely contributes to nematode containment and developmental arrest. Biochemical assays further demonstrated greater induction of defense-related enzymes, including peroxidase (POD), polyphenol oxidase (PPO), and superoxide dismutase (SOD), in the roots of resistant lines. Notably, SA levels increased significantly upon infection in all resistant roots, particularly in *C. metuliferus*, compared with the susceptible control (Barracuda F1). This elevated SA accumulation correlated with the upregulated expression of *PR1-1A*, *PR3*, and *LOX1*. Overall, resistance in the selected inbred lines is multifaceted, encompassing reduced nematode development, HR-associated necrosis, enhanced antioxidant enzyme activity, and coordinated SA-mediated gene upregulation. These responses most pronounced in *C. metuliferus* play a crucial role in mitigating root-knot nematode damage and provide valuable targets for breeding nematode-resistant cucumber genotypes.

**Supplementary Information:**

The online version contains supplementary material available at 10.1186/s12870-026-09586-4.

## Introduction

Cucumber (*Cucumis sativus* L.), one of the top ten vegetables globally, is highly susceptible to root-knot nematodes (RKN), which cause major economic losses in both open-field and protected cultivation, with greenhouse yield reductions of up to 88% [1, 13, 19, 21, 26, 52]. Although nematicides provide some level of control, their toxicity, high cost, and environmental risks necessitate alternative management strategies [38]. Host plant resistance is the most economical and environmentally sustainable approach for managing RKN [12, 14], however, commercially viable cucumber cultivars with high resistance to *Meloidogyne incognita* have historically been unavailable [28]. This has prompted research into wild relatives as resistance sources, with *Cucumis metuliferus* (African horned cucumber) and *Cucumis africanus* identified as promising resistant genotypes [32, 40, 44, 47, 49].

Resistance to RKN involves either pre- or post-penetration mechanisms. Pre-penetration resistance prevents invasion through physical barriers or root exudates that repel juveniles or lack attractants [12, 30]. Post-penetration resistance allows initial invasion but restricts nematode development via incompatible host responses [10], characterized by failed feeding-site establishment, often associated with a hypersensitive response (HR)-mediated cell death or poorly developed giant cells [35, 46]. Plant immunity against RKN is governed by PAMP-triggered immunity (PTI) and effector-triggered immunity (ETI) [8, 25, 39]. These immune responses activate reactive oxygen species (ROS) accumulation, phytohormone signaling (particularly salicylic acid), and transcriptional reprogramming of defense-related genes [18, 31, 37, 48]. In resistant *Cucumis* species, resistance typically involves impaired nematode reproduction, poor giant-cell development, or HR-mediated necrosis [12]. Although resistance in *C. metuliferus* and *C. africanus* has been documented, the underlying molecular mechanisms including specific resistance genes, defense signaling networks, and gene expression profiles remain largely uncharacterized [7, 33].

Given the global economic importance of RKN and the lack of resistant commercial cucumber cultivars, this study aimed to develop new *Cucumis* inbred lines resistant to *M. incognita*. Through a targeted breeding and selection program involving *C. sativus*, *C. sativus* var. *hardwickii*, and *C. metuliferus*, horticulturally promising homozygous resistant lines were generated. The resistance phenotype was characterized through morphological, histological, biochemical, and horticultural assessments under nematode infestation. Additionally, defense responses were investigated by analyzing salicylic acid levels and expression patterns of key innate immunity genes to elucidate the underlying resistance mechanisms.

## Material and method

### Experimental location and plant materials

#### Plant material and breeding program

This study was conducted to develop root-knot nematode-resistant inbred lines with superior horticultural traits from three *Cucumis* genotypes: *Cucumis sativus* var. *sativus* (Ames 20,089), *Cucumis sativus* var. *hardwickii* (PI 215589), and *Cucumis metuliferus* (PI 482442). The experiments were carried out from 2023 to 2025 under micro-plot (3 m × 3 m × 1 m) and greenhouse conditions. Micro-plots were filled with sterilized sandy clay soil (1:1 v/v) and planted with 20 plants per plot. The study was conducted at a private farm in the Abou Ghaleb region and at the Horticultural Research Institute, Agricultural Research Center, Egypt. The parental genotypes were obtained from the Cross-Pollinated Vegetable Crops Department, Horticultural Research Institute.

Self-pollination was performed on the plant materials (Table S1) to fix and accumulate desirable traits in the developing inbred lines. The breeding and selection program was initiated in September 2023 and conducted over five successive generations (F₂ to F₆) through artificial inoculation with *Meloidogyne incognita*. The F₂ generation served as the starting point for selection, and the F₆ generation was reached in Feb 2025. High selection pressure was applied to select inbred lines exhibiting both nematode resistance and desirable horticultural characteristics. Following the completion of selection, a comprehensive evaluation of horticultural traits, nematode resistance, biochemical parameters (POD, PPO, SOD), salicylic acid content, histopathological responses, and defense-related gene expression was conducted during the Summer–Autumn season of 2025.

#### Nematode culture and inoculum preparation

1. Source of *M. incognita*
  A pure culture of *Meloidogyne incognita* was originally obtained from infected cucumber roots collected from a commercial greenhouse in Berncht – El Aiat – Giza, Egypt, where severe root-knot symptoms were observed. The identity was confirmed as *M. incognita* based on perineal patterns of adult females [22] and morphometric analysis of second-stage juveniles (J2) [23].
2. Maintenance of the Culture
  The pure culture was maintained on eggplant (*Solanum melongena* cv. Laalae) in a greenhouse at 30 ± 3 °C. Ten-day-old eggplant seedlings were transplanted into 15-cm plastic pots filled with sterilized sandy clay soil (1:1 v/v) and inoculated with a single egg mass to maintain genetic homogeneity. This cycle was repeated every 2–3 months to maintain fresh, viable inoculum.
3. Extraction of Second-Stage Juveniles (J2)
  For inoculation, eggs and J2 were extracted from heavily galled roots of 60-day-old eggplant plants, with modifications. Roots were washed, chopped into 1–2 cm pieces, and blended in 0.5% sodium hypochlorite (NaOCl) for 1 min [16]. The suspension was poured onto a 200-mesh sieve (75 µm aperture) stacked over a 500-mesh sieve (25 µm aperture). The material collected on the 500-mesh sieve was rinsed thoroughly with tap water and placed on a Baermann funnel lined with tissue paper for 24 h to allow active J2 to migrate into clean water.
4. Viability and Hatching Assessment
  Viability of extracted J2 was assessed immediately before each inoculation using 0.05% methylene blue staining. A 0.1 mL aliquot of the J2 suspension was mixed with an equal volume of 0.05% methylene blue solution; dead J2 stained blue, while live J2 remained unstained. At least 100 J2 per batch were examined under a stereomicroscope (40 × magnification), and only batches with > 90% viability was used. Additionally, a hatching test was performed by incubating eggs in water at 25 °C for 7 days, and only batches with > 85% hatching rate were accepted.
5. Inoculum Preparation
  For each inoculation experiment, the final suspension was adjusted to 3000 J2 per mL using a Peters-type counting slide. Each plant received 10 mL of this suspension (3000 J2), applied into four holes around the base of the plant. The same nematode population was used throughout all breeding generations (F₂ to F₆) and the final evaluation experiments.

#### Homogeneity test

For each genotype, 150 plants were grown over five generations (F₂ to F₆). Individual plants with the best horticultural traits and *M. incognita* resistance were selected. The selected inbred lines were then sown to calculate the coefficient of variation (CV%) for key commercial traits: total yield (kg) and root gall number. Homogeneity among genotypes was assessed using CV, calculated as the experimental error standard deviation divided by the trial mean.

#### Selection stage

Seeds of Cucumis spp. were sown for five successive seasons in 3 × 3 × 1 m micro-plots filled with sterilized sandy clay soil (1:1 v/v). Four weeks after sowing, when seedlings had reached the 3–4 true leaf stage (approximately 25–28 days old), Each plant received 10 mL of nematode suspension containing 3000 M. incognita second-stage juveniles (J2s). Based on the soil volume and bulk density of the micro-plots (9 m^3^; ≈1.3 g/cm^3^), this corresponded to an inoculum density of approximately 5.13 J2s per kg of soil.. Plants were self-pollinated, and horticultural traits were recorded. At the end of each season, roots were uprooted, washed, and stained with acid fuchsin [16]. Galls, egg masses, and eggs per root were counted. Initial and final populations were recorded, and the reproduction factor (RF) was calculated as RF = FP/IP.

#### Evaluation stage

Seedlings of selected genotypes (three *C. sativus*, three *C. sativus* var. *hardwickii*, and five *C. metuliferus* promising inbred lines, along with the commercial hybrid Barracuda F₁ as a susceptible control) were transplanted 7 days before artificial inoculation. The experiment was conducted under greenhouse conditions at 25–35 °C Day/18–23 °C night, arranged in a randomized complete block design with three replicates. Each seedling was inoculated with 3000 *M. incognita* second-stage juveniles. At the end of the season, all nematode parameters were assessed as described above. Genotypes were evaluated for vegetative, flowering, fruit, and yield traits:

- Vegetative growth: main stem length (cm), plant fresh weight (g), internode length (cm), number of branches, number of leaves, and leaf area (cm^2^).
- Flowering traits: days to first female flower anthesis, female flowers per node, female flowers per plant, male flowers per plant, and sex ratio.
- Fruit characteristics: length (cm), diameter (cm), average fruit weight (g), and shape index.
- Yield: early yield (weight (kg) and fruit number per plant during the first two harvest weeks); total yield (total weight (kg) and total fruit number per plant).

#### Enzyme estimation

Seedlings of the inbred lines and the control (Barracuda F₁) were transplanted into 15 cm diameter pots filled with sterilized sandy clay soil (1:1 v/v); two weeks later, they were separately inoculated with 3000 infective second-stage juveniles (J2) of *M. incognita*. For enzyme estimation, root samples were collected at 3-, 15-, and 30-days post inoculation (dpi), homogenized in extraction buffer, and centrifuged at 12,000 rpm for 15 min at 4 °C to obtain crude enzyme extracts. POD activity was determined following Gayoso et al. [17] by spectrophotometrically monitoring the oxidation of guaiacol (at 470 nm).PPO activity was assayed according to Rocha and Morais [42] using a Shimadzu UV 1601 spectrophotometer, based on the enzymatic oxidation of phenolic substrates such as catechol or 4-methylcatechol, measured at 400–420 nm, where one unit corresponds to a 0.001 absorbance increase per minute per mL of enzyme. SOD activity was evaluated by measuring absorbance at 560 nm via the nitro blue tetrazolium (NBT) photochemical reduction method, where one unit is defined as the amount of enzyme required to inhibit NBT photoreduction by 50%, and specific activity was normalized against tissue weight and extract volume.

#### Endogenous salicylic acid (SA) content

Root samples were collected at 3 days post inoculation, immediately frozen in liquid nitrogen, ground to a fine powder, and 10 mg of tissue was extracted using methanol:chloroform (9:1, v/v). The extract was then derivatized with BSTFA at 120 °C for 1 h according to Forcat et al. [15]. For quantitative analysis, a small volume (1–2 µL) of the derivatized sample was injected (often in splitless mode) into the GC, where separation was achieved using a non-polar to mid-polar capillary column (e.g., DB-5ms, 30 m × 0.25 mm × 0.25 µm) with a temperature program (e.g., starting at 80 °C and ramping to 280 °C). The separated compounds were detected by mass spectrometry operating in selected ion monitoring (SIM) mode, which tracked only the characteristic ions of the salicylic acid derivative and an internal standard throughout the run. This approach, widely considered the benchmark for reliable SA quantification in plant-pathogen interaction studies, enabled precise determination of SA levels in the nematode-inoculated seedlings.

#### Histopathological analysis

Root samples infected with *M. incognita* were collected from resistant, moderately resistant, and susceptible genotypes at 30 dpi. For each genotype, three biological replicates were collected, with each replicate consisting of roots pooled from three individual plants. Root samples were fixed in formalin-acetic acid-alcohol (FAA) for at least 48 h. Dehydration was carried out using a graded series of n-butyl alcohol and ethanol. Roots were then infiltrated and embedded in paraffin wax at 52 °C for 5 days. Sections of the processed root portions were cut using a rotary microtome at a thickness of 13–15 µm. For each root system, 10 longitudinal and 10 cross-sections were examined (total of 20 sections per root system, 180 sections per genotype). Sections were stained with safranin and fast green and mounted in Canada balsam [9]. For each genotype, a minimum of 30 feeding sites (10 per biological replicate) were examined at 400 × magnification. All histological images were captured using a compound microscope (Olympus BX51, Japan) equipped with a digital camera. Scale bars and labels were added using ImageJ software.

#### RNA isolation and quantitative real-time PCR (qPCR) analysis

Root tissues were collected at 0-, 48-, and 72-h post-inoculation (hpi) for each treatment. For each time point and treatment, three biological replicates were collected, with each biological replicate consisting of roots pooled from three individual plants. All harvested samples were immediately flash-frozen in liquid nitrogen and stored at –80 °C until further processing.

Total RNA was extracted from 100 mg of ground root tissue using TRIzol™ reagent (Thermo Fisher Scientific, USA) according to the manufacturer's protocol. To eliminate residual genomic DNA contamination, the extracted RNA was treated with DNase I (Cat. No. EN0525, Thermo Scientific). RNA concentration and purity were assessed spectrophotometrically using a NanoDrop™ ND-1000 (Thermo Fisher Scientific), and RNA integrity was verified by electrophoresis on a 1.2% denaturing agarose gel. First-strand cDNA was synthesized from 1 µg of total RNA using SuperScript™ II Reverse Transcriptase (Cat. No. 18064014, Thermo Scientific) with oligo(dT) primers, following the manufacturer's instructions. Quantitative real-time PCR (qPCR) was performed using the Mx3000P QPCR System (Agilent Technologies, USA). The reaction mixture (15 µL final volume) consisted of 7.5 µL of 2 × SYBR® Green PCR Master Mix (Applied Biosystems), 0.5 µM of each gene-specific primer, and 2 µL of diluted cDNA. The thermal cycling protocol, adapted from Beaubois et al. [6], was as follows: initial denaturation at 95 °C for 10 min, followed by 40 cycles of 95 °C for 15 s and 60 °C for 1 min. A dissociation (melt) curve analysis was performed from 60 °C to 95 °C (with a ramp rate of 0.5 °C per 30 s) to confirm the amplification of single, specific products. Each qPCR run included non-template controls (NTC) for each primer pair. Gene-specific primers for *LOX1*, *PR1-1A*, and *PR3* (SA pathway genes) and the constitutive reference gene *Actin* are listed in Table S2. *Actin* was used as the reference gene for normalizing gene expression values, as its expression remained stable across all experimental conditions. Three independent technical replicates were used for gene expression analysis, and all reactions were performed in triplicate technical replicates for each biological replicate.

#### Experimental design and statistical analysis

The experiments were arranged in a randomized complete block design (RCBD) with three replicates. Each replicate consisted of 20 individual plants, resulting in a total of 60 plants per treatment. Data were analyzed by ANOVA using MSTAT-C software (Michigan State University, USA). Treatment means were compared using Fisher's least significant difference (LSD) test at *p* ≤ 0.05 according to Steel and Torrie [45].

## Results

### Selection and homogeneity estimation

Two key selection criteria total yield and number of nematode galls were applied across five inbreeding generations. Segregation occurred within each *Cucumis* species, and the selection progress for each species is summarized in Supplementary Tables S3, S4, and S5, showing a progressive shift toward resistant root gall index classes across generations. After five generations, *C. sativus* var. *sativus*, *C. sativus* var. *hardwickii*, and *C. metuliferus* produced three, three, and five inbred lines, respectively. In each generation, the best plants were selected for highest yield and effective RKN resistance.

**Fig. 1:**
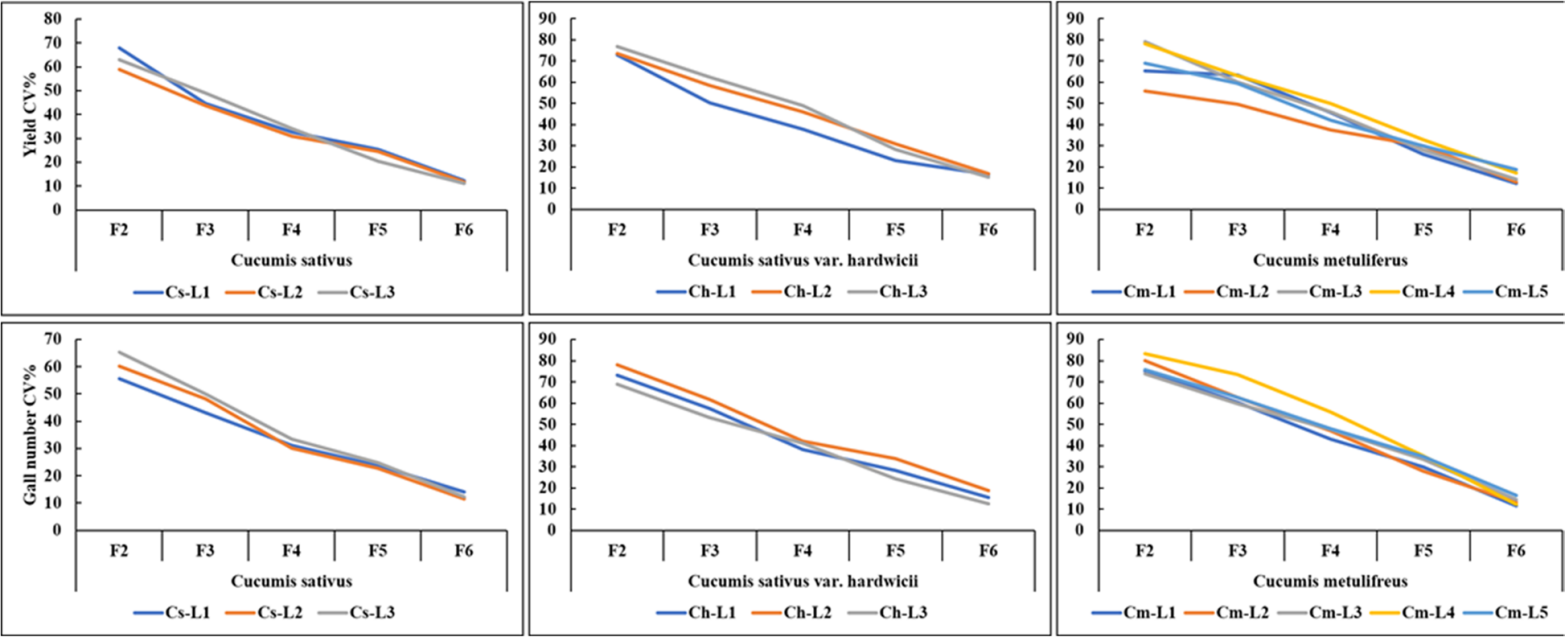
Coefficient of variation (CV%) trends for total yield and gall number and across five generations (F₂–F₆) of inbreeding in cucumber (*Cucumis sativus*), *Cucumis sativus* var. *hardwickii*, and *Cucumis metuliferus* inbred lines. Data are presented for three *C. sativus* lines, three *C. sativus* var. *hardwickii* lines, and five *C. metuliferus* lines. CV% values were calculated for each generation to assess the stabilization of phenotypic traits during inbreeding

The coefficient of variation (CV%) of F₂–F₆ populations was estimated, and homogeneity for the eleven selected inbred lines (highest yield and resistance) is shown in Fig. 1. For *C. sativus* var. *sativus*, homogeneity correlated positively with inbreeding. CV% for yield and gall number dropped from 68% and 65.3% in F₂ to 11% and 11.6% in F₆, respectively. For *C. sativus* var. *hardwickii*, CV% for both traits in F₂ ranged from 73–78% (high heterogeneity). In F₃, CV% ranged from 50–60% (still heterogeneous). F₄ CV% ranged from 49–38%, and F₅ ranged from 33–23%. Selection continued to F₆, where CV% reached 12.5% (galls) and 15.3% (yield), indicating high homogeneity. For *C. metuliferus*, F₂ CV% ranged from 83.2–55.8%, while F₆ CV% reached 11.6% (galls) and 12.4% (yield), confirming homogeneity. Notably, inbreeding did not reduce plant vigor or yield components over successive seasons.

### Horticultural evaluation

Data presented in Table 1 and Supplementary Table S6 indicate that the three inbred lines of *Cucumis sativus* var. *sativus* were evaluated for the most significant vegetative characteristics: main stem length (cm), plant fresh weight (g), internode length (cm), number of branches, leaf area (cm^2^), and number of leaves. The data showed significant differences among the three inbred lines for all six vegetative traits. Inbred line Cs-L1 exhibited the highest significant values for main stem length, plant fresh weight, and internode length. Inbred line Cs-L2 revealed the highest values with significant differences in number of branches and number of leaves, while leaf area showed the highest significant value in inbred line Cs-L3. Concerning *Cucumis sativus* var. *hardwickii*, inbred lines Ch-L2 and Ch-L3 exhibited the highest vegetative traits without significant differences between them, except for number of leaves, which showed the highest count in inbred line Ch-L2. Assessment of traits in horned cucumber (*Cucumis metuliferus*) revealed that inbred line Cm-L3 exhibited the highest significant values compared with other inbred lines for all studied traits, except for number of branches and number of leaves, which were superior in inbred line Cm-L1.

**Table 1:** Vegetative growth and flowering of selected *Cucumis* spp inbred lines under RKN, *M. incognita* infection

Genotypes	MST (cm)	PFW(g)	IL (cm)	NB	LA (cm^2^)	NL	NDFF	NM/plant	NFF/plant
*C. sativus*									
Cs-L_1_	370.0 a	365.1 a	7.6 a	2.9 b	339.8 b	82.0 b	24.6 b	2.1c	59.9 c
Cs-L_2_	262.7 c	214.7 c	5.1 c	3.9 a	307.4 c	106.7 a	28.7 a	3.1b	66.8 b
Cs-L_3_	313.0 b	249.2 b	6.1 b	2.2 c	524.1 a	70.9 b	26.03 ab	3.5a	74.6 a
LSD	17.6	30.3	0.45	0.63	31.3	13.3	3.4	0.3	2.5
SD	6.3	10.9	0.2	0.2	11.2	4.8	1.2	0.1	0.9
*C. sativus var. hardwickii*									
Ch-L1	243.0 b	280.0 b	4.0 b	12.5 a	226.0 b	157.0 c	65.0 b	123.0 a	36.0 c
Ch-L2	282.0 a	342.0 a	4.9 a	14.0 a	299.0 a	202.0 a	87.0 a	106.0 c	39.0 b
Ch-L3	300.0 a	303.0 ab	5.2 a	13.8 a	280.0 a	182.0 b	72.0 b	121.0 b	42.5 a
LSD	15.0	29.0	0.5	1.3	17.0	10.0	4.5	1.0	1.5
SD	5.5	10.5	0.18	0.5	6.0	3.6	1.6	0.4	0.55
*C. metuliferus*									
Cm-L_1_	367.0 a	431.0 b	4.0 c	142.0 d	20.7 a	257.0 a	77.0 c	129.0 b	38.0 b
Cm-L_2_	297.0 b	368.0 c	4.7 b	168.0 b	22.1 a	227.0 b	96.0 a	133.0 a	50.0 a
Cm-L_3_	379.0 a	458.0 a	5.9 a	184.0 a	17.4 b	220.0 b	81.0 c	112.0 e	35.0 c
Cm-L_4_	297.0 b	340.0 d	4.1 c	150.0 cd	17.4 b	157.0 c	88.0 b	118.0 c	26.5 e
Cm-L_5_	244.0 c	284.0 e	4.2 c	156.0 c	11.2 c	167.0 c	96.0 a	116.0 d	32.0 d
LSD	14.0	13.0	0.30	7.0	1.8	8.5	3.9	1.0	0.9
SD	6.0	5.6	0.13	3.0	0.8	3.7	1.7	0.44	0.38

MST (cm): main stem length, PFW(g): plant fresh weight, IL (cm): internode length, NB: number of branches, LA (cm^2^): leaf area, NL: number of leaves, NDFF: number of days to first female flower anthesis, NM/plant: number of male flowers/plant, NFF/plant: number of female flowers/plant

The findings in Tables 1, 2, and Supplementary Tables S6 and S7 revealed that among the selected *C. sativus* var. *sativus* lines, inbred line Cs-L1 significantly recorded the lowest number of male flowers and the fewest days to first female flower anthesis, indicating that it is the earliest-yielding inbred line. Furthermore, Cs-L1 produced the longest fruit, the heaviest fruit weight, and consequently the highest yield. Inbred line Cs-L3 exhibited the highest number of female flowers per node and the highest number of female flowers per plant. For C. sativus var. hardwickii, inbred line Ch-L1 recorded the earliest time to first female flower opening. In contrast, inbred line Ch-L3 exhibited the highest number of female flowers per plant, the longest fruit, the heaviest fruit weight, and the highest fruit shape index. Based on fruit characters, inbred line Ch-L3 showed the highest values for fruit length, fruit shape index, and fruit weight. For C. metuliferus, inbred line Cm-L2 showed the highest number of female flowers per plant, while inbred line Cm-L1 showed the fewest days to female flower opening. Inbred line Cm-L3 exhibited the longest fruit length, highest fruit shape index, and greatest fruit weight. Concerning early and total yield, inbred line Cm-L2 gave the highest values compared with other inbred lines.

**Table 2:** Fruit and yield component of selected *Cucumis* spp inbred lines under RKN, *M. incognita* infection

	**Fruit length (cm)**	**Fruit diameter (cm)**	**Fruit shape index**	**Fruit weight (g)**	**Early fruit number**	**Early fruit weight (g)**	**Total fruit number**	**Total fruit weight (kg)**
C. sativus								
Cs-L_1_	21.3 a	2.5 a	8.69 a	121.4 a	20.97 a	2496.7 a	63.03 a	7.65 a
Cs-L_2_	15.3 c	2.9 a	5.26 b	91.1 c	10.77 b	667.0 b	38.6 b	3.5 b
Cs-L_3_	17.8 b	2.4 a	7.6 a	100.6 b	18.3 a	1000.8 b	57.6 a	5.8 a
LSD	1.5	0.6	2.14	5.87	3.47	762.8	14.1	1.89
SD	0.55	0.23	0.77	2.1	1.25	274.7	5.2	0.68
*C. sativus var. hardwickii*								
Ch-L_1_	7.09 b	4.49 a	1.57 b	102.4 b	3.02 b	308.36 b	18.08b	1.85 b
Ch-L_2_	5.96 b	3.93 a	1.52 b	93.73 c	4.23a	396.63 a	25.43a	2.38 a
Ch-L_3_	9.26 a	3.99 a	2.3 a	122.3 a	2.7 b	334.06 b	16.4b	2.03b
LSD	1.16	ns	0.49	ns	0.43	47.84	2.61	0.29
SD	0.42	-	0.18	-	0.15	17.23	0.94	0.105
*C. metuliferus*								
Cm-L_1_	5.67 b	4.07 a	1.39 c	116.3 d	3.13 ab	363.7 c	18.77 b	2.18 d
Cm-L_2_	6.13 b	4.03 a	1.53 bc	158.3 b	4.77 a	756.8 a	28.6 a	4.54 a
Cm-L_3_	7.17 a	3.49 a	2.046 a	183.0 a	2.77 d	505.8 b	16.6 c	3.0407 b
Cm-L_4_	5.93 b	3.83 a	1.54 bc	147.7 bc	3.33 b	492.03 b	17.67 bc	2.607 c
Cm-L_5_	6.30 ab	3.50 a	1.77 ab	140.3 c	2.90 cd	407.0 c	15.0 d	2.1 d
LSD	0.94	0.62	0.36	11.46	0.26	58.78	1.43	0.32
SD	0.41	0.27	0.16	4.97	0.11	25.49	0.62	0.14

Table 2 and Supplementary Table S7 show that early and total yield, expressed as number and weight per plant, differed significantly across genotypes. For *C. sativus* var. *sativus*, inbred lines Cs-L1 and Cs-L3 recorded the highest fruit weight (7.65 and 5.8 kg/plant) and fruit numbers (63.03 and 57.6) without significant differences between them. Furthermore, yield values for C. sativus var. hardwickii were very high for inbred line Ch-L2, with significant differences, recording 2.37 kg/plant. Based on yield component characters, C. metuliferus inbred line Cm-L2 exhibited the highest values compared with the other inbred lines, recording 4.54 kg/plant and 28 fruits/plant, compared with 2–3 kg/plant and 15–18 fruits/plant for the other selected inbred lines.

### Reaction of selected Cucumis spp. Inbred Lines to M. incognita

**Fig. 2:**
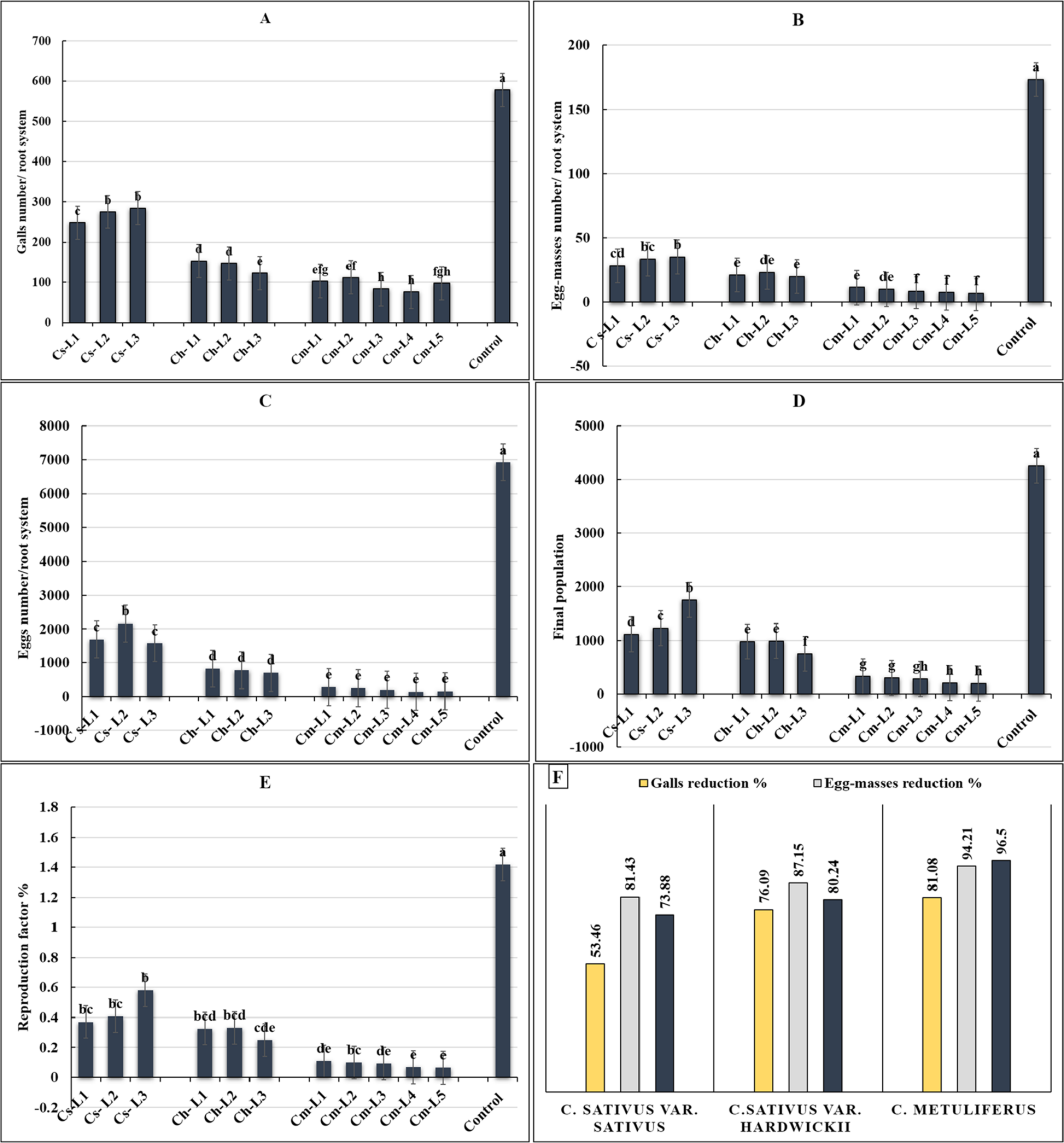
Resistance screening of selected *Cucumis* spp. inbred lines against the root-knot nematode *Meloidogyne incognita*. Panels **a–e** present the absolute values of nematode infection and reproduction parameters, including number of galls (a), egg-masses (b), eggs per root system (c), final population density (Pf, d), and reproductive factor (Rf = Pf/Pi, e). Panel **f** displays the percentage reduction in galls, egg-masses, eggs, and Pf for the resistant lines, calculated relative to the mean values of the susceptible control plants.. Different letters above bars indicate significant differences with LSD *P* ≤ 0.05

Figures 2a–e show significant differences in all nematode parameters among different inbred lines compared with the control. Control cucumber plants had the most infected roots, resulting in the greatest gall numbers of 555 and 545 per plant root system during the first and second seasons, respectively. All selected inbred lines from different species were more resistant to root-knot nematode than the control. The lowest gall numbers were observed in all selected *Cucumis metuliferus* inbred lines, particularly inbred line Cm-L4, where plants had the lowest gall counts of 75.29 and 72.40 per plant root system in the two seasons, respectively. Across both seasons, all C. metuliferus inbred lines exhibited the lowest egg mass numbers (6 egg masses) compared with other inbred lines and the control (156 egg masses). The *C. sativus* var. *hardwickii* inbred lines showed superiority in nematode resistance compared with *C. sativus* var. *sativus* inbred lines and the control across all nematode parameters.

The findings revealed that selected *C. sativus*, *C. metuliferus*, and *C. sativus* var. *hardwickii* inbred lines reduced fecundity, and egg counts declined compared with the control. In both seasons, egg deposition in control plants was 6412.7 and 6061.3 eggs per plant root system, respectively. Although the initial population was 3000 J2s, the final population values for all inbred lines were lower than the initial population, except for the susceptible control, which reached a final population exceeding the initial (4457 J2s). All inbred lines and the control showed significant variations in nematode reproduction parameters.

The data in Fig. 2f demonstrate a reduction in galls, egg masses, and eggs with various selected inbreds from different *Cucumis* species. The reduction percentages for galls, egg masses, and egg numbers increased to 81.08%, 94.21%, and 96.5%, respectively, in *C. metuliferus* inbred lines. Furthermore, the results illustrate the effectiveness of *C. sativus* var. *hardwickii* resistant inbred lines, with reduction percentages increasing to 76.09%, 87.15%, and 80.24%, respectively. Meanwhile, the reductions for *C. sativus* var. *sativus* were 53.46%, 81.43%, and 73.88%. These results illustrate the effectiveness of the resistant inbred line approach for recurrent nematode control. Gall number decrease was observed in all eleven selected inbreds but was most pronounced in *C. metuliferus* selected inbred lines.

### Measurement of POD, PPO, and SOD activities

**Fig. 3:**
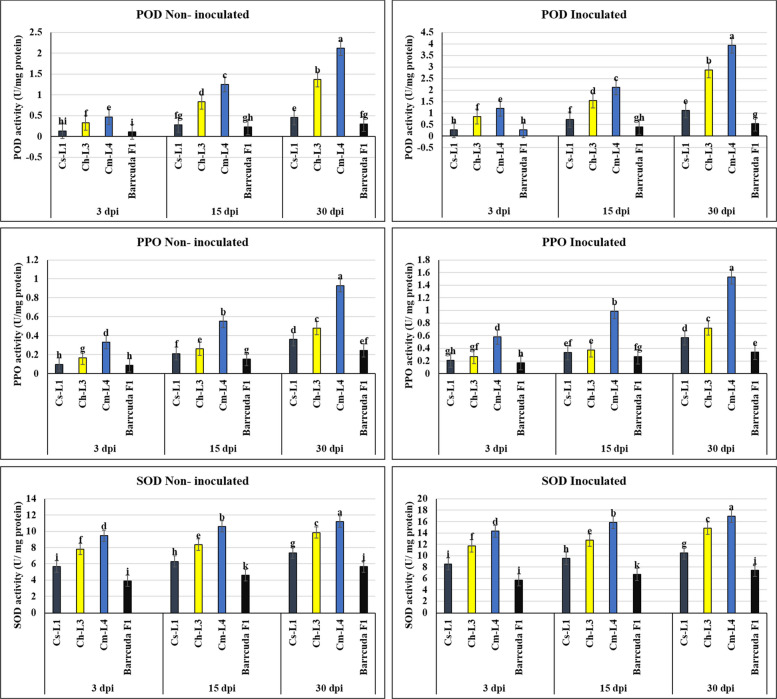
Time-course changes in the activities of defense-related antioxidant and oxidative enzymes in roots of selected resistant *Cucumis* spp. inbred lines compared with the susceptible control following inoculation with *Meloidogyne incognita*. Panels **a**, **b**, and **c** represent the enzymatic activities of peroxidase (POD), polyphenol oxidase (PPO), and superoxide dismutase (SOD), respectively, measured at various time points post-inoculation. Different letters above bars indicate significant differences with LSD *P* ≤ 0.05

Analysis of antioxidant enzyme activities revealed significant differences among genotypes across all time points (Fig. 3a–c). In general, the three most resistant inbred lines *C. sativus* inbred Cs-L1, *C. sativus* var. *hardwickii* inbred Ch-L3, and *C. metuliferus* inbred Cm-L4 were tested for activities of peroxidase (POD), polyphenol oxidase (PPO), and superoxide dismutase (SOD) compared with the susceptible control (F1 Barracuda) following *M. incognita* infection.

Across both seasons, POD activity increased with plant age and was further enhanced by nematode inoculation, with *C. metuliferus* consistently showing the highest levels, followed by *C. sativus* var. *hardwickii*, then *C. sativus*. In *C. metuliferus*, POD concentration rose from 0.47 to 2.1 µg/g fresh weight (FW) in non-inoculated plants and from 2.12 to 3.94 µg/g FW in inoculated plants between 3 and 30 days of age. A similar pattern was observed in *C. sativus* var. *hardwickii*, with POD levels increasing from 0.32 to 1.3 µg/g FW in non-inoculated roots and from 0.84 to 2.86 µg/g FW in inoculated roots over the same period. In the *C. sativus* resistant inbred Cs-L1, POD concentrations ranged from 0.13 to 0.46 µg/g FW in non-inoculated plants and from 0.27 to 1.12 µg/g FW in inoculated plants. No significant differences were detected between this line and the susceptible control at 3 days, but significant differences emerged at 15 and 30 days. At 30 days, *C. metuliferus* exhibited the greatest fold increases relative to the control, with POD activities 6.99-fold (non-inoculated) and 7.16-fold (inoculated) higher. In comparison, *C. sativus* var. *hardwickii* showed increases of 4.5-fold and 5.2-fold, respectively, while the *C. sativus* resistant inbred showed 1.52-fold and 2.03-fold increases.

PPO content was positively correlated with both plant age and infection status across all genotypes. *C. metuliferus* again exhibited the highest PPO levels at all sampling times. At 30 days, PPO activities in *C. metuliferus* were 3.8-fold (non-inoculated) and 4.5-fold (inoculated) higher than the control. *C. sativus* var. *hardwickii* showed increases of 1.97-fold and 2.12-fold, respectively, while the *C. sativus* resistant inbred showed increases of 1.49-fold and 1.66-fold.

SOD activity increased progressively from 3 to 30 days post-inoculation in all resistant lines. *C. metuliferus* recorded the highest SOD values at 30 days (11.2 in non-inoculated and 16.9 in inoculated roots), whereas the susceptible control exhibited the lowest values at 3 days (3.9 and 5.7, respectively). At 30 days, SOD activities in *C. metuliferus* were 1.97-fold and 2.27-fold higher than the control for non-inoculated and inoculated roots, respectively. *C. sativus* var. *hardwickii* showed increases of 57% and 50%, while the *C. sativus* resistant inbred showed 1.29-fold and 1.41-fold increases.

Overall, the activities of POD, PPO, and SOD were consistently higher in resistant roots than in susceptible roots, with peak levels observed at 30 days post-inoculation. Among the resistant genotypes, *C. metuliferus* inbred Cm-L4 exhibited the highest antioxidant enzyme activities across most sampling stages, followed by *C. sativus* var. *hardwickii* inbred Ch-L3 and *C. sativus* inbred Cs-L1.

### Histological response to nematode infection

**Fig. 4:**
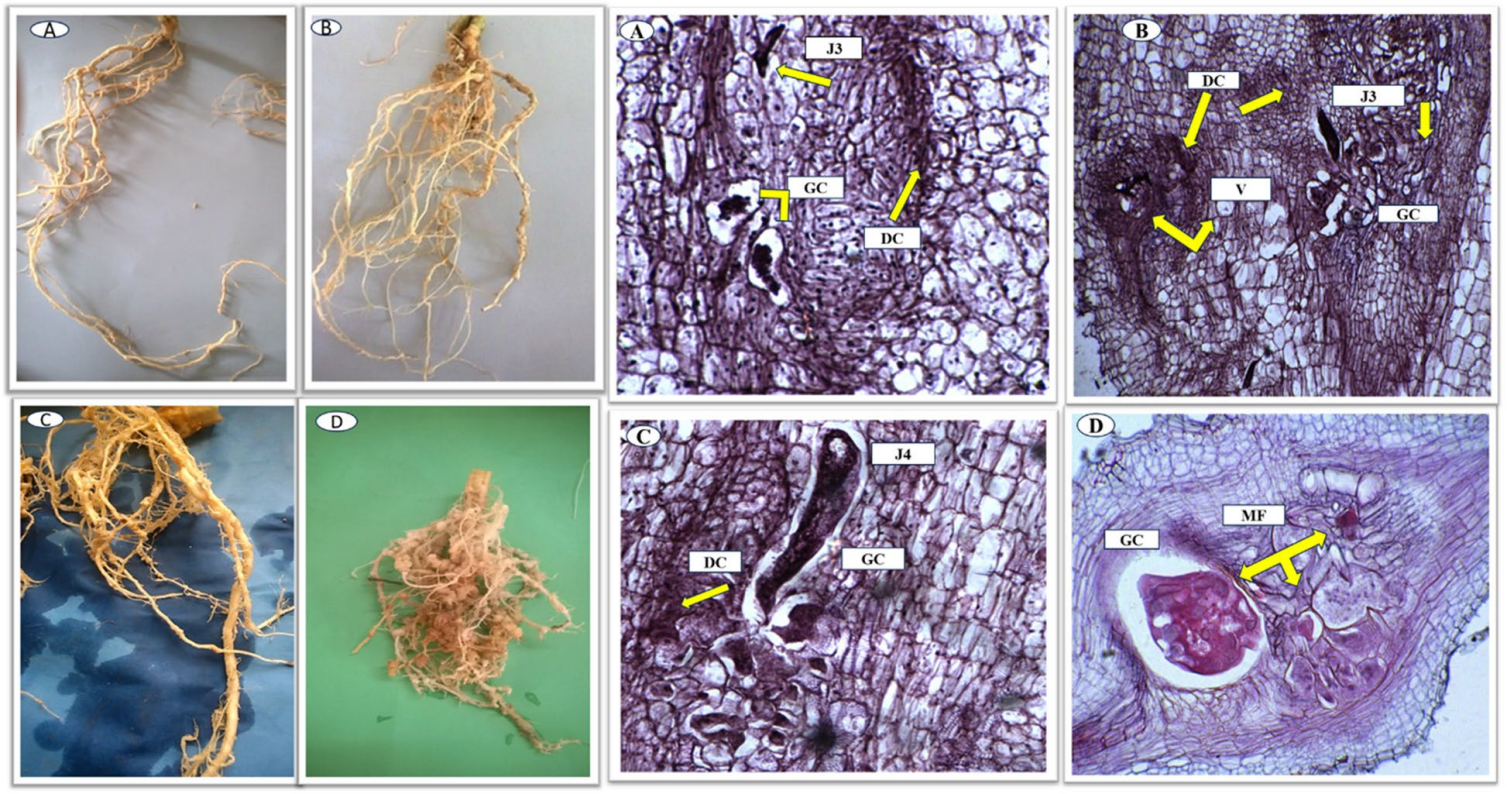
Histological sections and symptom severity of *M. incognita*-infected roots from (**a**) *C. metuliferus*, (**b**) *C. sativus* var. *hardwickii*, (**c**) *C. sativus* var. *sativus*, and (**d**) uninoculated control at 35 dpi. Annotations: GC, giant cell; DC, dead cell; NC, necrotic cell; V, vacuole; J3, third-stage juvenile; MF, mature female

Morphological and histopathological comparisons at 35 days post-inoculation revealed distinct differences between the susceptible control (*C. sativus* 'Barracuda F1') and the resistant inbred lines (Cs-L1, Ch-L3, and Cm-L4) (Fig. 4a–d). In susceptible roots, numerous well-developed giant cells with dense cytoplasm, enlarged nuclei, and thick cell walls formed adjacent to nematode heads, with no evidence of necrosis or physical barriers.

In contrast, resistant lines exhibited impaired feeding site development: giant cells were limited in number (2–3 per site), and in *C. metuliferus* Cm-L4, both normal and poorly developed giant cells were observed, with small giant cells associated with immature nematodes. Hypersensitive necrosis occurred in giant cells adjacent to nematodes in resistant roots, whereas no such response was seen in the susceptible control. Furthermore, giant cells in resistant lines displayed large vacuoles (occupying > 60% of the cellular area), thin cell walls, and cytoplasmic degeneration, suggesting premature collapse, while susceptible roots maintained progressive giant cell enlargement. Nematode development was also restricted. In *C. sativus* var. *hardwickii* Ch-L3, second-stage juveniles (J2s) remained abundant (comprising > 80% of the total nematode population), with limited progression to J3 or J4 stages (< 10%). In *C. metuliferus* Cm-L4, J2s predominated (> 70%), while later-stage juveniles were rarely observed. The proportion of necrotic feeding sites was significantly higher in resistant lines (68.5%) than in susceptible controls (8.7%), consistent with a hypersensitive response (HR)-like mechanism. While these observations are suggestive of HR-like programmed cell death, histological evidence alone does not provide definitive proof of HR.

### Effect of resistance on defense phytohormone (SA) content

**Fig. 5:**
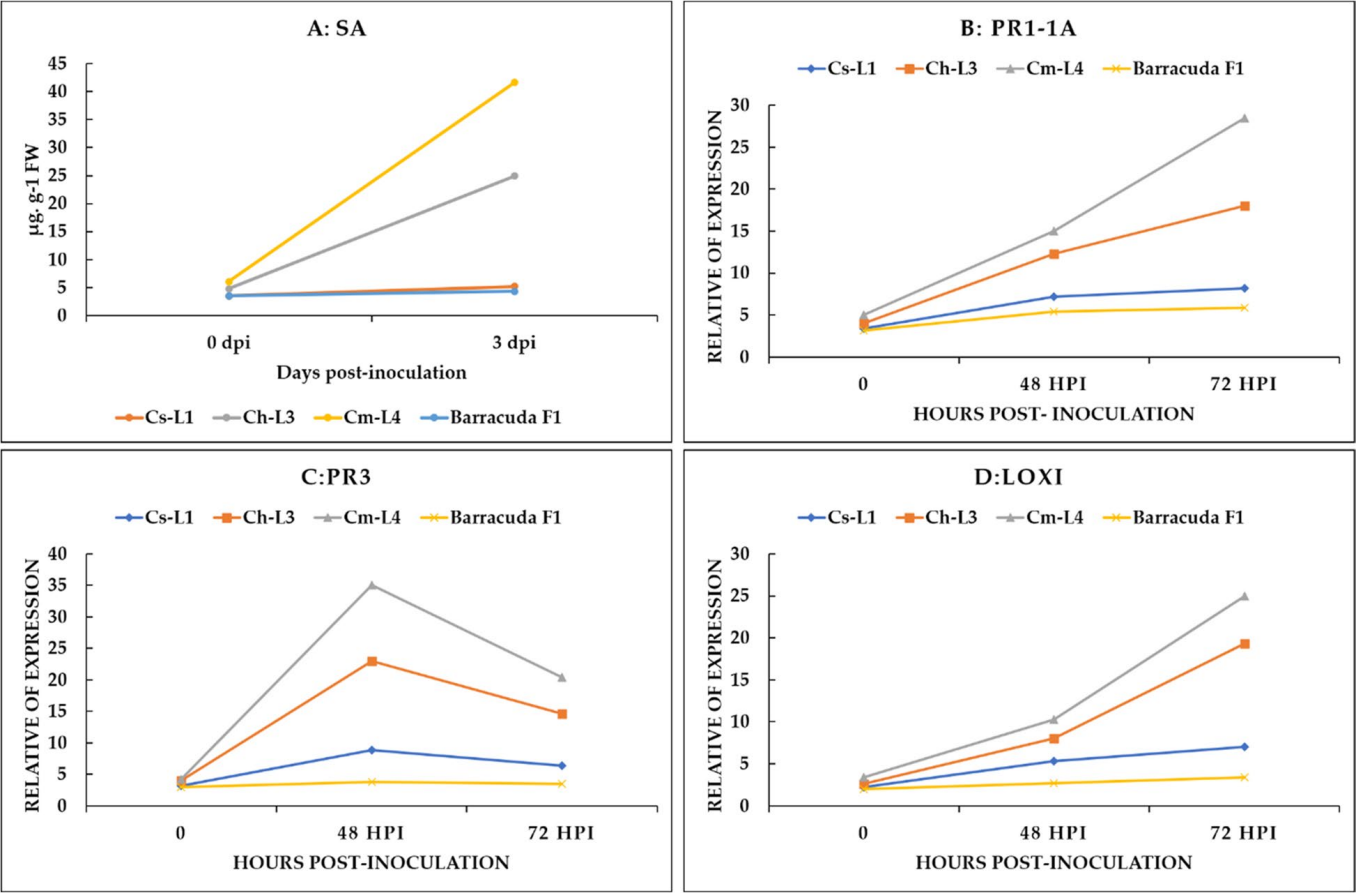
Biochemical and molecular responses in roots of selected resistant *Cucumis* spp. inbred lines compared with the susceptible control following inoculation with *Meloidogyne incognita*. Panel **a** shows salicylic acid (SA) content. Panels **b**–**d** present the relative transcript levels (determined by RT-qPCR) of the defense-related genes **b** *PR-1A*, **(c)** *PR-3* (chitinase), and **d** *LOX1* at the indicated time points post-inoculation

To investigate whether resistant inbred lines influence levels of defense-related phytohormones, salicylic acid (SA) was quantified in all resistant lines and the susceptible control before and after inoculation with *M. incognita* (Fig. 5a). At 3 days post-inoculation (dpi), SA was significantly upregulated in the roots of resistant genotypes compared with the control. Specifically, SA levels increased by 9.68-fold in *C. metuliferus* (Cm-L4) and by 5.79-fold in *C. sativus* var. *hardwickii* (Ch-L3) relative to the control. In contrast, the resistant *C. sativus* inbred line Cs-L1 exhibited only a modest increase, with SA levels not exceeding 1.2-fold above the control following nematode infestation.

### Effect of Resistance on expression levels of sa-related genes

Analysis of defense-related gene expression in roots revealed genotype-dependent temporal patterns (Fig. 5b–d). In *C. metuliferus* Cm-L4, expression levels of *LOX1* and *PR1-1A* were significantly higher than in the susceptible control at 72 h post-inoculation (hpi), with fold changes of 7.35 and 5.8, respectively. Expression of *PR3* in this line peaked at 48 hpi (9.2-fold) and remained elevated at 72 hpi (5.8-fold). Similarly, *C. sativus* var. *hardwickii* Ch-L3 showed substantial increases at 72 hpi for *LOX1* (5.67-fold) and *PR1-1A* (3.67-fold), while *PR3* reached its highest level at 48 hpi (6.05-fold). In contrast, the *C. sativus* inbred line Cs-L1 exhibited only modest increases at 72 hpi (*LOX1*: 2.05-fold; *PR1-1A*: 1.6-fold; *PR3*: 1.8-fold). Notably, for all resistant inbred lines, *PR3* expression was significantly downregulated at 72 hpi compared with the corresponding 48 hpi values, indicating a transient induction pattern.

## Discussion

Infection by *M. incognita* caused significant reductions in growth and yield attributes across cucumber cultivars, with the magnitude of reduction corresponding to their level of susceptibility. The global reliance on synthetic nematicides has raised environmental and health concerns, driving the search for sustainable alternatives [13]. Recent comprehensive reviews have further emphasized advanced breeding strategies and the genetic architecture underlying agronomic traits in cucurbits, reinforcing the potential of resistance breeding as a durable solution [50],Balagoni [4]. In this context, breeding for resistance to root-knot nematodes (RKN) has emerged as a promising strategy to enhance plant tolerance to both biotic and abiotic stresses. The present study was conducted to evaluate the efficacy of resistant cucumber inbred lines against *M. incognita* and to elucidate the physiological and molecular mechanisms associated with their protective effects.

The results demonstrate that the breeding program significantly improved vegetative growth parameters including main stem length, plant fresh weight, internode length, number of branches, leaf area, and number of leaves in resistant inbred lines infested with *M. incognita* compared with susceptible controls [12]. These findings are consistent with previous reports indicating that nematode resistance promotes growth by enhancing water and nutrient uptake and modulating phytohormonal balance [3, 7, 20]. The growth suppression observed in susceptible plants is attributable to high nematode population densities, which impair root function and limit nutrient and water absorption [5, 38]. Importantly, resistant inbred lines not only restored growth but also improved flowering traits and fruit yield both in number and weight corroborating earlier findings by Kayani et al. [29] and El-Remaly [12]. These beneficial effects extended across multiple growth parameters, underscoring the value of resistance as an integrated management tool.

Through five generations of self-pollination coupled with concurrent selection for horticultural traits and nematode resistance, a set of promising inbred lines was developed. Among these, lines Cs-L1, Ch-L2, Cm-L1, and Cm-L2 exhibited superior horticultural characteristics, while Ch-L3, Cm-L4, and Cm-L5 displayed the lowest levels of nematode infection, as measured by gall numbers, egg masses, and eggs per root system. The successful development of lines combining acceptable agronomic performance with high resistance underscores the feasibility of introgressing nematode resistance into cultivated backgrounds, a goal that aligns with contemporary breeding frameworks for cucurbits [4]. These findings confirm that selected inbred lines express a high level of resistance to *M. incognita*, consistent with earlier reports documenting resistance to *Meloidogyne* species in *Cucumis* germplasm [20, 47, 51]. Recent work demonstrated that root volatiles in a resistant *C. metuliferus* line included (methoxymethyl)-benzene, which repelled *M. incognita* J2s, whereas certain compounds in susceptible lines acted as attractants. These findings align with our observed reduction in J2 penetration and point to root chemistry as a key determinant of host suitability.

For nematodes that successfully penetrated, post-penetration resistance was associated with impaired feeding site development and delayed nematode maturation. Quantitative histopathological analysis (30 root segments per line, examined at 14, 21, and 28 dpi) revealed that in resistant lines, the mean number of giant cells per feeding site was significantly lower (2.1 ± 0.3 in *C. metuliferus* Cm-L4 vs. 6.8 ± 0.7 in the susceptible control at 21 dpi). Giant cells in resistant roots were also smaller in mean diameter (18.4 ± 2.1 µm vs. 42.6 ± 3.5 µm in the control) and more frequently exhibited large vacuoles, thin cell walls, and cytoplasmic degeneration. In many cases, giant cells appeared collapsed or devoid of cytoplasm, consistent with previous reports in resistant *Cucumis* accessions [14]. Consequently, fewer nematodes reached the adult stage in resistant lines (e.g., 1.8 ± 0.4 adult females per section in *C. metuliferus* Cm-L4 vs. 12.5 ± 1.2 in the control), with most individuals remaining at the J2 or J3 stage. These quantitative observations suggest that *C. metuliferus* and *C. sativus* var. *hardwickii* may function as poor hosts that do not sustain the nutrient demands required for nematode completion of the life cycle [11].

A central point of discussion in the literature concerns whether resistance in *Cucumis* involves a hypersensitive response (HR). Early studies, including those by Walters et al. [47], reported no evidence of necrosis or HR in resistant *Cucumis* species, attributing resistance solely to the failure of giant cells to develop properly. In the present study, we observed localized cell necrosis and browning adjacent to nematode heads in some sections of resistant lines. Quantitatively, resistant lines showed an average of 4.2 ± 0.6 necrotic cells per feeding site at 21 dpi, compared with 0.3 ± 0.1 in the susceptible control. Vacuolation preceding cell death was noted in these areas. These features morphologically resemble a hypersensitive response,however, because we did not perform functional tests (e.g., HR-specific marker expression or reactive oxygen burst assays), we refer to this as an HR-like reaction rather than definitive HR. Similar observations of multivacuolated, collapsed giant cells and necrosis have been reported in more recent studies [14, 51]. As noted in the present work, the genetic background plays a critical role in shaping the interaction with *M. incognita*. Regardless of the presence or absence of visible necrosis, the functional outcome impaired giant cell function and arrested nematode development was consistently associated with resistance across genotypes [41].

Histological examination of resistant lines revealed localized necrosis and cellular degeneration at nematode feeding sites, accompanied by the presence of dead or degenerating nematodes. These features are consistent with a hypersensitive response (HR)-like cell death mechanism, which has been implicated in resistance to sedentary endoparasitic nematodes in several plant species [40]. However, we acknowledge that histological evidence of necrosis alone is not conclusive proof of HR. Definitive confirmation would require additional cytological and molecular markers, such as autofluorescence, callose deposition, hydrogen peroxide accumulation, or expression of HR-associated marker genes. Thus, while the data are suggestive of HR-like cell death, further studies are needed to confirm the involvement of classic programmed cell death pathways in the resistance response of these inbred lines.

Resistant inbred lines exhibited significantly higher activities of defense-related antioxidant enzymes, including peroxidase (POD), polyphenol oxidase (PPO), and superoxide dismutase (SOD), compared with the susceptible control [24, 36], with *C. metuliferus* consistently showing the greatest increases. POD activity, in particular, was associated with resistance, peaking at 30 days post-inoculation. Elevated POD activity is thought to contribute to defense through multiple mechanisms, including the generation of reactive oxygen species, oxidation of phenolics, and reinforcement of cell walls via lignin and suberin deposition [2, 37]. The consistent correlation between high POD activity and resistance supports its potential utility as a marker for screening in breeding programs, pending independent validation. Notably, Jia et al. [24] demonstrated that enhanced antioxidant capacity, when combined with jasmonate-mediated cucurbitacin biosynthesis, effectively bolstered cucumber resistance against necrotrophic pathogens a mechanism that may similarly contribute to the resistance phenotypes observed here against sedentary nematodes.

Phytohormone profiling revealed that salicylic acid (SA) was modestly but significantly elevated in resistant roots at 3 days post-inoculation (dpi), with the highest accumulation observed in *C. metuliferus* (approximately 9.68-fold increase) and *C. sativus* var. *hardwickii* (approximately 5.79-fold increase) compared with the susceptible control. This SA accumulation was accompanied by the induced expression of pathogenesis-related (PR) genes, including *PR1-1A* and *PR3*. However, a more consistent molecular signature of resistance across the selected lines was the upregulation of *LOX1* (2- to fourfold at 48–72 hpi). *LOX1* is a key enzyme in the jasmonate (JA) biosynthesis pathway, and its upregulation was correlated with activation of JA-dependent defenses. The critical role of lipoxygenases in mediating plant responses to biotic stress particularly through JA signaling is well established [27, 34], and our data are consistent with this paradigm. In contrast, SA pathway markers showed variable or non-significant changes across lines. Therefore, while SA-mediated signaling may contribute to the resistance response, our data indicate that JA pathway activation (as suggested by *LOX1* upregulation) is more consistently associated with effective resistance against *M. incognita* in these *Cucumis* lines. SA may play a secondary or modulating role, potentially acting in concert with JA [43], rather than serving as the primary defense pathway. In susceptible plants, both SA accumulation and *PR* gene expression were markedly lower, further supporting a link between integrated phytohormone signaling and effective resistance. Although a modest increase in endogenous SA was detected at 3 dpi in some resistant lines, the more consistent molecular signature was the upregulation of *LOX1*, a jasmonate pathway marker. This suggests that JA-mediated defense responses, rather than SA-dependent signaling, are predominantly associated with *M. incognita* resistance in the selected *Cucumis* inbred lines. SA may contribute to basal resistance or act in concert with JA, but our data do not support a primary role for SA. This is the first report associating *LOX1* upregulation with reduced nematode galling and impaired giant cell development in *C. metuliferus* and *C. sativus* var. *hardwickii*-derived inbred lines. While previous studies focused on SA or antioxidant enzymes alone, the work simultaneously profiles histology (with quantitative cell counts), enzyme activities, and phytohormone-related gene expression, revealing that JA-linked responses correlate more consistently with resistance than SA accumulation in these genetic backgrounds further reinforcing the pivotal role of lipoxygenase-mediated pathways in biotic stress resilience [34].

The resistant inbred lines developed in this study combine acceptable horticultural traits with reduced nematode reproduction. These lines may serve as valuable genetic resources for introgression breeding (transferring resistance loci into commercial cucumber cultivars; Balagoni [4] or as rootstocks for grafting. The correlative markers identified (elevated POD activity at 30 dpi, *LOX1* upregulation at 48–72 hpi) can aid in marker-assisted screening, but they require independent validation in breeding populations before routine application. Field trials across multiple seasons and locations are necessary to confirm durability and agronomic performance, as virulent nematode populations could potentially arise.

In summary, the multifaceted resistance associated with the selected *Cucumis* inbred lines comprising reduced J2 penetration, impaired giant cell development, an HR-like reaction (localized necrosis), elevated antioxidant enzyme activity, and coordinated phytohormone signaling was correlated with reduced nematode reproduction and development. The absence of reported virulent nematode populations capable of overcoming resistance in *C. metuliferus* [14] suggests that this resistance may be more durable, but this remains to be tested. Introgression of resistance genes from *C. metuliferus* into cultivated lines, or its direct use as a rootstock, presents a potential strategy for sustainable nematode management, pending field validation.

## Conclusions

In conclusion, this study demonstrates that selected *Cucumis* inbred lines resist *M. incognita* through a multifaceted defense strategy encompassing reduced J2 penetration, impaired giant cell development, localized hypersensitive-like necrosis, elevated antioxidant enzyme activities, and transcriptional shifts, with LOX1 (JA pathway) upregulation correlating more consistently with resistance than SA. This work provides the first integrated linkage of these histological, enzymatic, and molecular markers in *Cucumis*-RKN interactions, notably revealing an HR-like reaction in *C. metuliferus* that contrasts with earlier reports. The resistant lines Cm L_4_ and Ch L_3_ hold practical value for introgression breeding or grafting, while elevated POD (30 dpi) and LOX1 (48–72 hpi) represent candidate markers for pre-screening pending independent validation. Importantly, all mechanistic inferences remain correlative; functional validation and multi-environment field trials are requisite to establish causation and confirm durability. Overall, this study lays a robust foundation for future mechanistic investigations and applied breeding programs aimed at sustainable nematode management.

## Supplementary Information

Supplementary Material 1.

Supplementary Material 2.

## Data Availability

The datasets used and/or analysed during the current study are available from the corresponding author on reasonable request.
