# Supplementary material for: Multi-trait assessment of Meloidogyne incognita resistance in developed Cucumis spp. inbred lines: horticultural, physiological, biochemical, and histological insights
Source: BMC Plant Biol. 2026 Aug 5;26:1307. doi: 10.1186/s12870-026-09586-4 (PMC13439878; doi:10.1186/s12870-026-09586-4)
Supplement: Supplementary file 1 [file 12870_2026_9586_MOESM1_ESM.docx]

**Supplementary Table 1.** The list of the *Cucumis* spp. genotypes used in the breeding program.

| **No.** | **Scientific name** | **Accession** | **Origin** | **Source** |
| --- | --- | --- | --- | --- |
| 1. | *Cucumis sativus var. sativus* | Ames 20089 | Egypt | USAD |
| 2. | *Cucumis sativus var. hardwickii* | PI 215589 | India | USDA |
| 3. | *Cucumis metulifeurs* | PI 482442 | Zimbabwe | USDA |
