# Supplementary material for: Multi-trait assessment of Meloidogyne incognita resistance in developed Cucumis spp. inbred lines: horticultural, physiological, biochemical, and histological insights
Source: BMC Plant Biol. 2026 Aug 5;26:1307. doi: 10.1186/s12870-026-09586-4 (PMC13439878; doi:10.1186/s12870-026-09586-4)
Supplement: Supplementary file 2 [file 12870_2026_9586_MOESM2_ESM.docx]

**S Table 1. The list of the *Cucumis* spp. genotypes used in the breeding program.**

| **No.** | **Scientific name** | **Accession** | **Origin** | **Source** | **Nematode resistance** |
| --- | --- | --- | --- | --- | --- |
| 1. | *Cucumis sativus var. sativus* | Ames 20089 | Egypt | USDA | SR |
| 2. | *Cucumis sativus var. sativus* | Beit alpha cv |  |  | S |
| 3. | *Cucumis sativus var. hardwickii* | PI 215589 | India | USDA | MR |
| 4. | *Cucumis sativus var. hardwickii* | Sumter cv |  |  | S |
| 5. | *Cucumis metuliferus* | PI 482442 | Zimbabwe | USDA | HR |
| 6. | *Cucumis metuliferus* | PI 482450 | Zimbabwe |  | S |
| 7. | *Cucumis sativus var. sativus* | Barracuda F_1_ |  |  | S |

**S. Table 2. Primers used for RT-qPCR analysis**

| **ID** | **Forward Primer** | **Reverse Primer** |
| --- | --- | --- |
| LOX1 | AGGACTTCCTGTTCCATTGGA | TGGAGGAAATTCCTTAAGACGA |
| PR1 1A | GTTGGGCCGATTGAGTGG | GCATCTCACTTTGGCACATCC |
| PR3 (chitinase) | TTCAATCATCACCAAGCAAAG | TCCGCAACTTCTACAGTCAA |
| Actin | GCCCTCCCTCATGCCATTCT | TCGGCAGTGGTGGTGACAT |

**Supplementary table 3. Reaction of Individual plant distribution of F_2_, F_3_, F_4_, F_5_, and F_6_ *C. sativus var. sativus* compared with the resistant and susceptible parents for resistance to root-knot nematode, *M. incognita* based on root gall index**. (F1: Ames 20089x Beit alpha cv).

| **Generations** | **Root gall index^*^classes** | | | | | |
| --- | --- | --- | --- | --- | --- | --- |
|  | **1** | **2** | **3** | **4** | **5** | **tested plants** |
|  | 0 = immune (I) | 1-10%(HR) | 11-20%(MR) | 21-40%(SR) | ˃ 40%(S) |  |
| F_2_ | 0 | 14 | 25 | 42 | 69 | 150 |
| F_3_ | 0 | 10 | 13 | 27 | 50 | 100 |
| F_4_ | 0 | 19 | 23 | 17 | 32 | 90 |
| F_5_ | 0 | 34 | 22 | 21 | 19 | 95 |
| F_6_ | 0 | 20 | 30 | 35 | 15 | 100 |
| Ames 20089 | 0 | 10 | 16 | 14 | 0 | 40 |
| Barrcuda F_1_ | 0 | 0 | 0 | 7 | 33 | 40 |

0=Immune.

1-10%	= Highly resistant.

11-20% = Moderately resistant.

21-40% = Slightly resistant.

>40%	= Susceptible

S table 4. Reaction of Individual plant distribution of F_2_, F_3_, F_4_, F_5_, and F_6_ *C. sativus var. hardwickii* compared with the resistant and susceptible parents for resistance to root-knot nematode, *M. incognita* based on root gall index. (F_1_: PI 215589x Sumter cv)

| **Generations** | **Root gall index^*^classes** | | | | | |
| --- | --- | --- | --- | --- | --- | --- |
|  | **1** | **2** | **3** | **4** | **5** | **tested plants** |
|  | 0 = immune (I) | 1-10%(HR) | 11-20%(MR) | 21-40%(SR) | ˃ 40%(S) |  |
| F_2_ | 1 | 29 | 25 | 31 | 64 | 150 |
| F_3_ | 2 | 14 | 15 | 27 | 42 | 100 |
| F_4_ | 0 | 19 | 31 | 17 | 28 | 95 |
| F_5_ | 0 | 34 | 26 | 16 | 19 | 95 |
| F_6_ | 3 | 47 | 32 | 10 | 8 | 100 |
| PI 215589 | 4 | 25 | 11 | 0 | 0 | 40 |
| Sumter cv | 0 | 0 | 1 | 6 | 33 | 40 |

S table 5. Individual plant’s reaction distribution of F_2_, F_3_, F_4_, F_5_, and F_6_ *C. metulifeus* compared with the resistant and susceptible parents for resistance to root-knot nematode, *M. incognita* based on root gall index. (F_1_: PI 482442x PI 482450)

| **Generations** | **Root gall index^*^classes** | | | | | |
| --- | --- | --- | --- | --- | --- | --- |
|  | **1** | **2** | **3** | **4** | **5** | **tested plants** |
|  | 0 = immune (I) | 1-10%(HR) | 11-20%(MR) | 21-40%(SR) | ˃ 40%(S) |  |
| F_2_ | 4 | 20 | 25 | 30 | 41 | 120 |
| F_3_ | 6 | 24 | 20 | 27 | 23 | 100 |
| F_4_ | 6 | 25 | 31 | 20 | 18 | 100 |
| F_5_ | 7 | 34 | 26 | 11 | 12 | 90 |
| F_6_ | 11 | 45 | 30 | 4 | 0 | 90 |
| PI 482442 | 4 | 29 | 7 | 0 | 0 | 40 |
| PI 482450 | 0 | 0 | 0 | 5 | 35 | 40 |

S. Table 6: Vegetative growth and flowering of selected *Cucumis* spp inbred lines under normal non- inoculated soil

| **Genotypes** | **MST (cm)** | **PFW(g)** | **IL (cm)** | **NB** | **LA (cm^2^)** | **NL** | **NDFF** | **NM/plant** | **NFF/plant** |
| --- | --- | --- | --- | --- | --- | --- | --- | --- | --- |
| ***C. sativus*** | | | | | | | | | |
| **Cs-L_1_** | 462.0 a | 510.0 a | 9.1 a | 3.5 b | 475.0 b | 106.0 b | 32.0 b | 3.2 c | 86.0 c |
| **Cs-L_2_** | 329.0 c | 300.0 c | 6.1 c | 4.7 a | 430.0 c | 138.0 a | 37.5 a | 4.5 b | 96.0 b |
| **Cs-L_3_** | 391.0 b | 349.0 b | 7.3 b | 2.7 c | 733.0 a | 92.0 b | 34.0 ab | 5.0 a | 107.0 a |
| **LSD** | 14.0 | 24.0 | 0.40 | 0.50 | 28.0 | 10.0 | 2.8 | 0.25 | 2.0 |
| **SD** | 5.0 | 8.5 | 0.15 | 0.18 | 10.0 | 3.8 | 1.0 | 0.09 | 0.7 |
| ***C. sativus var. hardwickii*** | | | | | | | | | |
| **Ch-L1** | 186.7 b | 200.4 b | 3.2 b | 9.4 a | 161.9 b | 120.9 c | 50.3 b | 94.7 a | 25.9 c |
| **Ch-L2** | 217.3 a | 244.2 a | 3.95 a | 10.6 a | 213.4 a | 155.8 a | 67.2a | 81.3 c | 27.9 b |
| **Ch-L3** | 230.7 a | 216.6 ab | 4.2 a | 10.5 a | 199.9 a | 140.4 b | 55.2 b | 93.3 b | 30.4 a |
| **LSD** | 18.9 | 36.5 | 0.6 | 1.6 | 20.8 | 12.6 | 5.5 | 1.3 | 1.9 |
| **SD** | 6.8 | 13.2 | 0.2 | 0.6 | 7.5 | 4.5 | 1.9 | 0.5 | 0.7 |
| ***C. metuliferus*** | | | | | | | | | |
| **Cm-L_1_** | 282.7 a | 308.1 b | 3.217 c | 129.7 d | 14.8 a | 197.8 a | 59.3 c | 99.7 b | 27.0 b |
| **Cm-L_2_** | 228.3 b | 262.6 c | 3.8 b | 152.8 b | 15.8 a | 174.5 b | 74.2 a | 102.5 a | 35.7 a |
| **Cm-L_3_** | 291.8 a | 327.3 a | 4.7 a | 167.7 a | 12.4 b | 169.4 b | 62.4 c | 85.97 e | 24.9 c |
| **Cm-L_4_** | 228.7 b | 243.0 d | 3.3 c | 136.3 cd | 12.4 b | 120.7 c | 67.7 b | 91.3 c | 18.9 e |
| **Cm-L_5_** | 187.7 c | 202.7 e | 3.4 c | 141.7c | 8.0 c | 128.3 c | 74.3 a | 89.0 d | 22.9 d |
| **LSD** | 17.3 | 16.5 | 0.36 | 8.75 | 2.31 | 10.8 | 4.77 | 1.24 | 1.09 |
| **SD** | 7.5 | 7.1 | 0.16 | 3.79 | 1.03 | 4.7 | 2.07 | 0.54 | 0.47 |

**MST (cm):** main stem length**, PFW(g):** plant fresh weight**, IL (cm):** internode length**, NB:** number of branches**, LA (cm^2^):** leaf area**, NL:** number of leaves**, NDFF:** number of days to first female flower anthesis**, NM/plant:** number of male flowers/plant**, NFF/plant:** number of female flowers/ plant.

**S. Table 7.** Fruit and yield component of selected *Cucumis* spp inbred lines under non inoculated soil

|  | **Fruit length (cm)** | **Fruit diameter (cm)** | **Fruit shape index** | **Fruit weight (g)** | **Early fruit number** | **Early fruit weight (g)** | **Total fruit number** | **Total fruit weight (kg)** |
| --- | --- | --- | --- | --- | --- | --- | --- | --- |
| **C. sativus** | | | | | | | | |
| **Cs-L_1_** | **25.6 a** | **2.8 a** | **10.43 a** | **150 a** | **25.5 a** | **3000 a** | **75.6 a** | **9.2 a** |
| **Cs-L_2_** | **18.4 c** | **3.2 a** | **6.31 b** | **110 c** | **13.0 b** | **800 b** | **46.3 b** | **4.2 b** |
| **Cs-L_3_** | **21.4 b** | **2.7 a** | **9.12 a** | **125 b** | **23.5 a** | **1200 b** | **69.1 a** | **8.0 a** |
| **LSD** | **1.2** | **0.48** | **1.71** | **4.7** | **2.8** | **610** | **11.3** | **1.5** |
| **SD** | **0.44** | **0.18** | **0.62** | **1.68** | **1.0** | **220** | **4.2** | **0.54** |
| ***C. sativus var. hardwickii*** | | | | | | | | |
| **Ch-L1** | **8.9 b** | **4.8 a** | **1.88 b** | **125 b** | **3.7 b** | **385 b** | **22.6 b** | **2.4 b** |
| **Ch-L2** | **7.5 c** | **4.2 a** | **1.82 b** | **115 c** | **5.3 a** | **496 a** | **31.8 a** | **3.0 a** |
| **Ch-L3** | **11.6 a** | **4.3 a** | **2.76 a** | **150 a** | **3.6 b** | **418 b** | **21.0 b** | **2.55 b** |
| **LSD** | **0.93** | **0.5** | **0.39** | **12** | **0.34** | **38.3** | **2.09** | **0.23** |
| **SD** | **0.34** | **-** | **0.14** | **-** | **0.12** | **13.8** | **0.75** | **0.084** |
| ***C. metuliferus*** | | | | | | | | |
| **Cm-L_1_** | **7.09 b** | **4.5 a** | **1.74 b** | **145 e** | **3.76 c** | **455 d** | **23.5 b** | **2.73 d** |
| **Cm-L_2_** | **7.66 b** | **4.4 a** | **1.91 b** | **198 b** | **5.72 a** | **946 a** | **35.8 a** | **5.68 a** |
| **Cm-L_3_** | **8.96 a** | **3.8 a** | **2.56 a** | **228 a** | **3.32 d** | **632 b** | **20.8 d** | **3.80 b** |
| **Cm-L_4_** | **7.41 b** | **4.2 a** | **1.93 b** | **185 c** | **4.00 b** | **615 b** | **22.5 c** | **3.26 c** |
| **Cm-L_5_** | **7.88 b** | **3.9 a** | **2.21 b** | **174 d** | **3.48 d** | **509 c** | **18.8 e** | **2.63 d** |
| **LSD** | **0.75** | **0.5** | **0.29** | **9.2** | **0.21** | **47.0** | **1.14** | **0.256** |
| **SD** | **0.33** | **0.22** | **0.13** | **4.0** | **0.09** | **20.4** | **0.50** | **0.112** |
